# A hypothetical trivalent epigenetic code that affects the nature of human ESCs

**DOI:** 10.1371/journal.pone.0238742

**Published:** 2020-09-10

**Authors:** Yasuhisa Ishikawa, Kenta Nakai

**Affiliations:** 1 Department of Computational Biology and Medical Sciences, the University of Tokyo, Kashiwa-shi, Chiba, Japan; 2 Human Genome Center, the Institute of Medical Science, the University of Tokyo, Tokyo, Japan; Macau University of Science and Technology, MACAO

## Abstract

It has been suggested that DNA methylation can work in concert with other epigenetic factors, leading to changes in cellular phenotypes. For example, DNA demethylation modifications producing 5-hydroxymethylcytosine (5hmC) are thought to interact with histone modifications to influence the acquisition of embryonic stem cell (ESC) potency. However, the mechanism by which this occurs is still unknown. Thus, we systematically analysed the co-occurrence of DNA and histone modifications at genic regions as well as their relationship with ESC-specific expression using a number of heterogeneous public datasets. From a set of 19 epigenetic factors, we found remarkable co-occurrence of 5hmC and H4K8ac, accompanied by H3K4me1. This enrichment was more prominent at gene body regions. The results were confirmed using data obtained from different detection methods and species. Our analysis shows that these marks work cooperatively to influence ESC-specific gene expression. We also found that this trivalent mark is relatively enriched in genes related with immunity, which is a bit specific in ESCs. We propose that a trivalent epigenetic mark, composed of 5hmC, H4K8ac and H3K4me1, regulates gene expression and modulates the nature of human ESCs as a novel epigenetic code.

## Introduction

Epigenetic processes underlying cell differentiation and development strictly regulate the transcriptome machinery to specify gene expression patterns, which greatly influences the determination of cell fates. One epigenetic marker, 5-hydroxymethylcytosine (5hmC), is highly accumulated in embryonic stem cells and induced pluripotent stem cells as an oxidised form of DNA methylation [1–3], which implicates its functional importance in the pluripotency of these cells.

Recently, the concept of an “epigenetic code” was proposed [4–6]. This code corresponds to the genetic code and refers to a set of epigenetic marks specifying different phenotypes in different cells. It was defined in a previous study as follows: “The epigenetic code is the sum of epigenetic modifications and controls in a particular cell type that do not alter the underlying DNA sequence, including chemical changes in DNA, chromatin modifiers and non-coding RNA in eukaryotic cells. It is mainly defined by DNA (de)methylation and histone modifications” [4]. Therefore, the epigenetic code must include the co-occurrence of various factors in the same region and the triggering of cell-specific gene expression, resulting in the development of a certain cell-specific phenotype.

As an example, it is known that H3K4me3 and H3K27me3 co-occur at promoters in embryonic stem cells (ESCs) to form “bivalent” regions and that the downstream genes involved in development are maintained in a poised state; thus, these epigenetic markers regulate ESCs [7, 8]. Furthermore, the interplay between DNA (de)methylation and histone modifications has been characterised experimentally in mammals. For example, polycomb repressive complex 2 (PRC2) recruits TET proteins to keep DNA hypomethylated at bivalent promoters in mouse ESCs [9, 10]. Additionally, at enhancers, pioneer transcription factors cooperate with TET proteins to produce other epigenetic modifiers and establish monomethylation of H3K4 to increase chromatin accessibility [11, 12]. These factors can all be considered part of the epigenetic code. However, they are only a part of the whole code, which is far from being fully elucidated.

The present study was performed to discover a new candidate epigenetic marker that influences pluripotent stem cells such as ESCs. We performed a comprehensive search of the combination of 5hmC, which is abundant in ESCs, and histone modifications by surveying public data on human ESCs (hESCs) and mouse ESCs.

It has been reported that the distribution of 5hmC in the genome is similar to that of a histone modification, H3K4me1, which affects transcriptional activation [13]. We confirmed this finding and additionally found a striking similarity between the distribution of 5hmC and that of H4K8ac, which also affects transcriptional activation. Furthermore, we confirmed that the co-occurrence of 5hmC, H3K4me1 and H4K8ac is correlated with ESC-specific gene expression.

By applying a nonnegative matrix factorisation (NMF) algorithm, we found that the trivalent epigenetic mark is enriched in the gene body and in promoter regions of genes, some of which are related to ESC-specific natures, such as immunity. Here, we propose that the co-occurrence of these epigenetic factors specifies ESC-specific gene expression and confers the properties of hESCs.

## Results

### Genome-wide distribution of DNA and histone modifications

**Fig 1:**
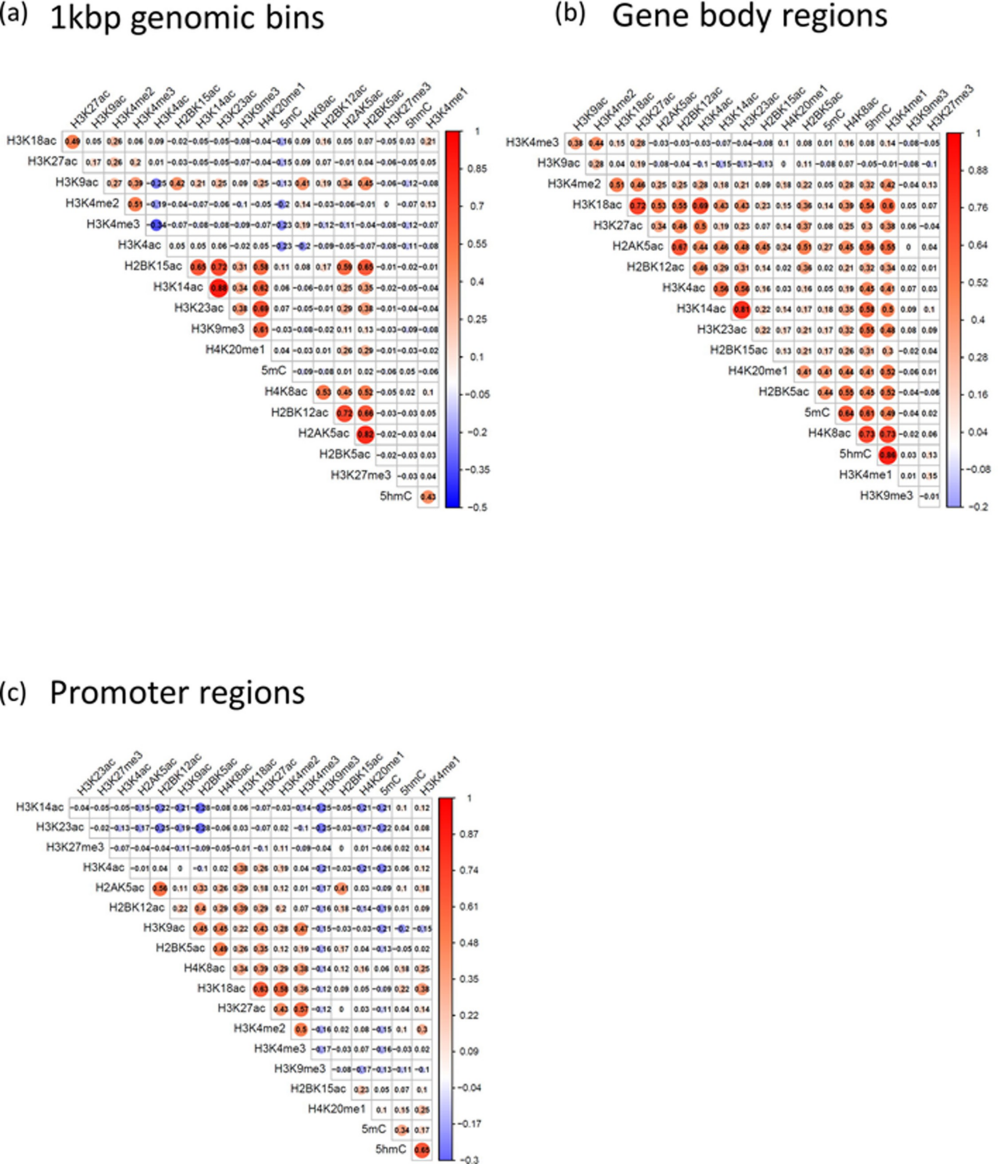
Correlation coefficients based on the intensities of epigenetic factors in hESC. The normalised mapped read count in each peak is assigned to each gene and is set as the factor intensity for that gene. The values of the figures are Pearson’s correlation coefficients between 19 factors (DNA and histone modifications) using the intensities of whole genes. (a) Correlation coefficients based on intensities per 1 kilobases (b) per gene body and (c) per promoter (promoter regions are defined as -5000 bp to 5000 bp from the TSS).

To characterise the epigenetic landscape in hESCs, we analysed large-scale public next-generation sequencing datasets containing information on 17 histone modifications, 5mC, and 5hmC (S1 Table). We first identified peak regions (false discovery rate (FDR) < 0.001) from the datasets and then calculated the normalised read count (i.e., intensity) of each peak. Next, we measured Pearson’s correlation coefficient (PCC) values of the intensities between all the possible pairs of epigenetic modifications in 1kbp genomic bins, gene body regions, and promoter regions (Fig 1).

Correlation analysis revealed a slight genome-wide co-enrichment tendency between modifications of the same type (Fig 1A); the methylation and acetylation of histones and their cognate modifications (e.g., H3K4me2 and H3K4me3; H3K9me3 and H4K20me1; H3K14ac and H3K23ac) had relatively high positive PCC values. Meanwhile, 5hmC showed a moderate correlation with H3K4me1 (PCC = 0.43), supporting the results of a previous study [13].

Interestingly, the degree of positive PCC values in the 1-kbp bins (Fig 1A) increased when only gene body regions were considered (Fig 1B). This implies that co-enrichment (of DNA and histone modifications) contributes to the regulation of gene expression in ESCs. In particular, co-enrichment between 5hmC and H4K8ac was more prominent (PCC = 0.73) at gene body regions. Although the co-enrichment of H4K8ac with DNA methylation decreased at promoter regions, it still remained positive (Fig 1C). These results indicate that 5hmC exists not only with H3K4me1 (PCC = 0.86 at gene body), which was already known [13], but also with H4K8ac at genic regions.

The statistical significance of these data was confirmed using other datasets obtained using different detection methods or species (S1 Fig). The PCC between the intensity of 5hmC according to TAB-seq and that according to hMeDIP-seq was high (PCC = 0.89). In addition, the intensity of 5hmC according to TAB-seq was highly correlated with that of H3K4me1 (PCC = 0.78) and H4K8ac (PCC = 0.69). In mouse ESCs, the PCC values were also high, albeit not as high as in hESCs (0.65 for 5hmC and H3K4me1, 0.54 for 5hmC and H4K8ac). This result shows that the correlations are not artefacts. In summary, certain pairs of histone and DNA modifications tend to co-occur in the same genomic loci. In particular, 5hmC significantly coexisted with the active histone modifications H4K8ac as well as H3K4me1, especially at gene body regions in hESCs.

### Enrichment of the trivalent domain in differentially expressed genes

**Fig 2:**
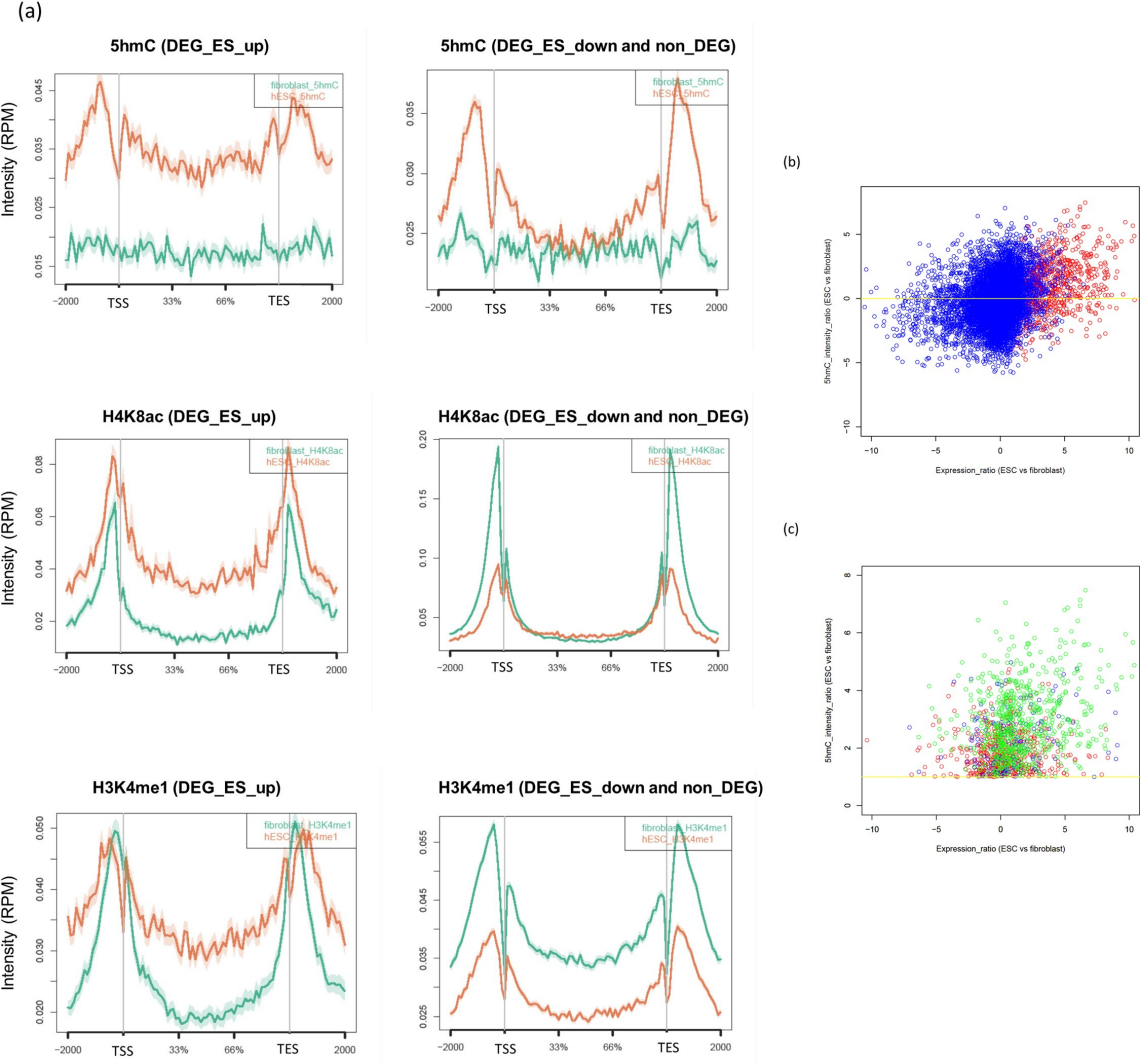
Distribution of reads for 5hmC, H4K8ac, and H3K4me1 around gene regions. (a) The difference of average distributions of each factor in the gene region. Orange lines are on hESC and green lines are on fibroblast. The y-axis indicates intensities (normalised read count), and the figures show the average distributions of reads on whole genes. (b) The scatter plots of genes between their expression ratio and the 5hmC intensity ratio. X-axis indicates gene expression ratio (ESC / fibroblast) in log2 scale. Y-axis indicates 5hmC intensity ratio (ESC / fibroblast) in log2 scale. The red circles show DEG_ES_up genes, while the blue circles show DEG_ES_down and non_DEG genes. (c) The scatter plot of genes between their expression ratio and the 5hmC intensity ratio with different number of marks. X-axis indicates gene expression ratio (ESC / fibroblast) in log2 scale. Y-axis indicates 5hmC intensity ratio (ESC / fibroblast) in log2 scale. Red circles represent genes which show only 5hmC enrichment, blue circles represent genes which show 5hmC and H3K4me1 enrichment only, and green circles represent genes which show the trivalent enrichment. All genes show 5hmC enrichment in ESCs (fold change over 1, as shown by yellow line).

To interrogate the possibility that the proposed trivalent domain (i.e., 5hmC, H3K4me1, and H4K8ac) regulates gene expression in ESCs, we first identified differentially expressed genes (DEGs) between hESCs and fibroblasts (S2 Fig); 2888 genes were upregulated (DEG_ES_up), 2141 genes were downregulated (DEG_ES_down), and 20,660 genes showed no clear differences in expression (non_DEG) in hESCs compared to fibroblasts (>2 |FC| and < 0.05 FDR). We then profiled the intensity distribution of the trivalent domain around gene regions for each of these three gene groups. We observed that rather than DEG_ES_down and non_DEG genes, DEG_ES_up genes in hESCs show overall enrichment for each trivalent epigenetic factor (Fig 2A). To investigate this tendency more precisely, scatter plots for each gene are shown (Fig 2B and 2C). Although DEG_ES_up genes (red circles in Fig 2B) do not show clear correlation between gene expression ratio and the intensity ratio of 5hmC. However, if we distinguish genes with the number of the marks (namely, red circles for genes with the 5hmC mark only, blue circles for genes with 5hmC and H3K4me1 only, and green circles for those with the three marks), we can recognise somewhat clear tendency that genes with more marks tend to show higher expression ratio as well as higher hydroxymethylation ratio. This indicates that the factors, when combined, have a more distinct effect on gene activation.

### Identification of trivalent enrichment patterns influencing gene regulation

**Fig 3:**
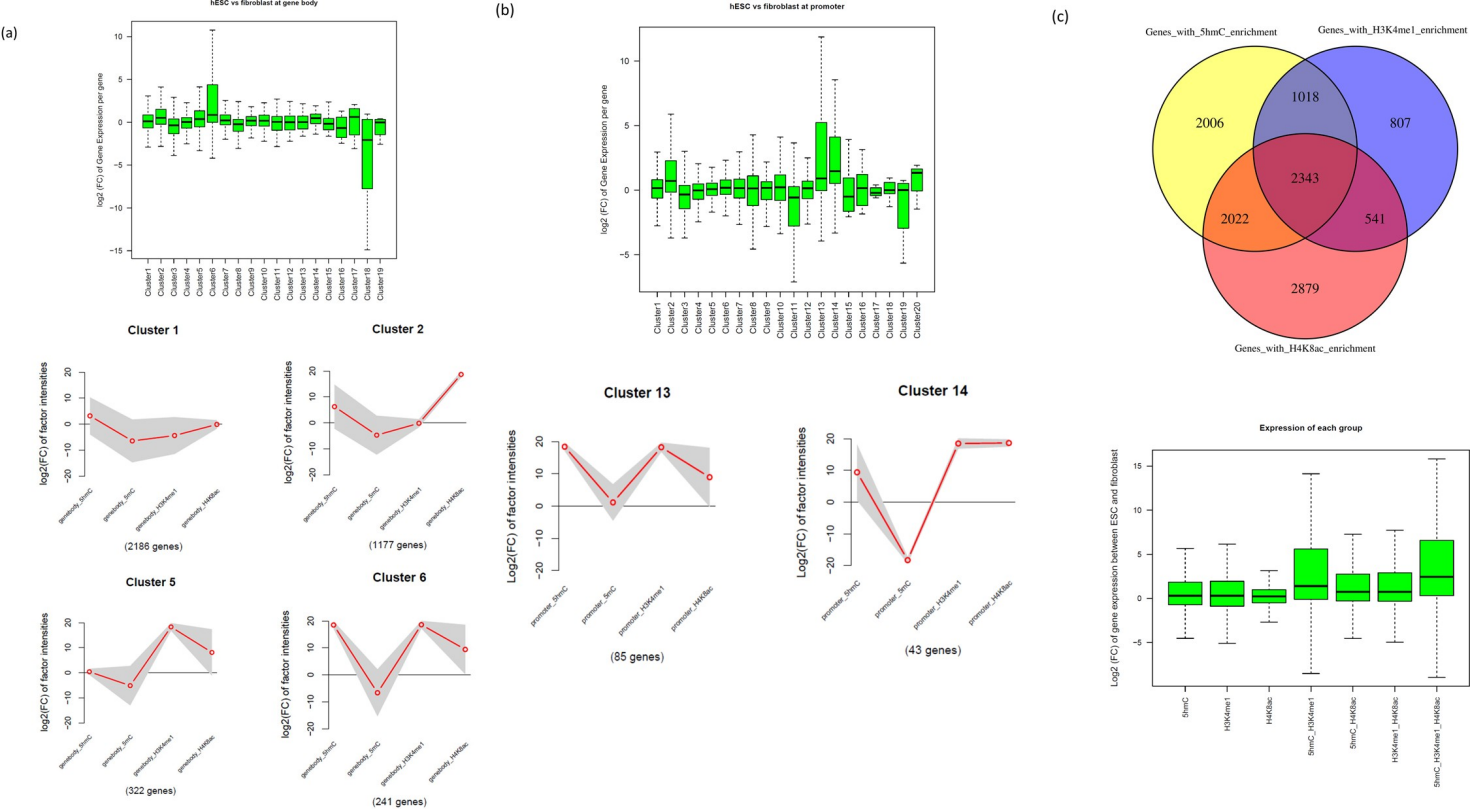
Trivalent enrichment patterns influencing gene regulation. (a) The upper figure shows the relationship between changes in gene expression and combinations of change in the epigenetic factor intensities at the gene body. Whole genes are classified to 19 gene groups based on the combinations. The y-axis shows logarithm of fold change in gene expression between hESCs and human fibroblasts. A positive value indicates that gene expression in hESCs is higher than in fibroblasts and vice versa for negative values. The lower figure shows combinations of change in the epigenetic factor intensities. The y-axis shows the logarithm of fold change in factor intensities between hESCs and human fibroblasts. A positive value indicates that the intensity of the factor in hESCs is higher than that in fibroblasts and vice versa for negative values. (b) Promoter data. (c) The necessity for the mark to be trivalent. The upper figure shows groups of genes enriched with each factor. A gene is regarded as enriched if the fold change of intensity for each factor is greater than 2 between hESC and fibroblast at gene body. The lower figure shows the Log2 (FC) value of gene expression among the seven gene groups in the above figure. For example, "5hmC" on the left end shows a gene group in which only 5hmC is enriched (corresponding to the gene group colored with yellow in the above figure).

To investigate the correlation between ESC-specific gene expression and the co-occurrence of 5hmC, H3K4me1 and H4K8ac, we performed hierarchical clustering analysis. Based on fold change (FC) values between hESCs and human fibroblasts, we characterised each gene according to the enrichment (intensity) pattern (vector) of 5hmC, 5mC, H3K4me1 and H4K8ac at its body. As a result, whole genes were classified into 19 clusters exhibiting (dis)similar expression between the two cell types (Fig 3A).

There was no clear distinction between cells in any of the four modifications in the majority of the gene body regions (i.e., 2186 genes in Cluster 1, as shown in Fig 3A); however, the H4K8ac modification was over-enriched in a large cluster in 1507 gene body regions (i.e., Cluster 2 in Fig 3A; see also Cluster 7 and Cluster 9 in S3 Fig). This result suggests the importance of H4K8ac marks in ESCs. Notably, H4K8ac over-enrichment co-occurred with 5hmC marks in hESCs, as shown by correlation analysis (Fig 1), corresponding to Cluster 6 (241 genes); these marks are part of the trivalent modification. Furthermore, this pattern [5hmC (+); 5mC (-); H3K4me1 (+); and H4K8ac (+)] was linked with relatively high gene expression levels in hESCs (boxplot in Fig 3A). The same pattern was also observed at promoter regions (Cluster 13 and Cluster 14 in Fig 3B). It is noteworthy that *NANOG*, a pluripotent stem cell marker gene, was contained in Cluster 6 in the gene body data (Fig 3A) and Cluster 13 in the promoter data (Fig 3B); *POU5F1* (Oct3/4) was classified in Cluster 14 in the promoter data (Fig 3B).

In contrast, the genes of Cluster 18, which are characterised by the pattern 5hmC (+), 5mC (+), H3K4me1 (-), and H4K8ac (-), showed repressive expression in hESCs (S3 Fig). This can be interpreted to mean that the methylations in the gene body are not sufficient for the activation of these genes. Similarly, two patterns [5hmC (+), 5mC (-), H3K4me1 (-), and H4K8ac (+) in Cluster 2; 5hmC (-), 5mC (-), H3K4me1 (+), and H4K8ac (+) in Cluster 5; Fig 3A] showed a steady-state degree of expression in the cells. These also suggest the importance of the trivalent modification. We also investigated the necessity for the mark to be trivalent as shown in Fig 3C. In the lower figure, the genes with trivalent enrichment (5hmC_H3K4me1_H4K8ac) show the greatest change in gene expression ratio between ESCs and fibroblasts. And the median is the farthest from zero. This shows that the epigenetic code proposed in this study needs to be trivalent rather than monovalent or bivalent.

### Systematic identification of genes marked by a defined set of epigenetic factors

**Fig 4:**
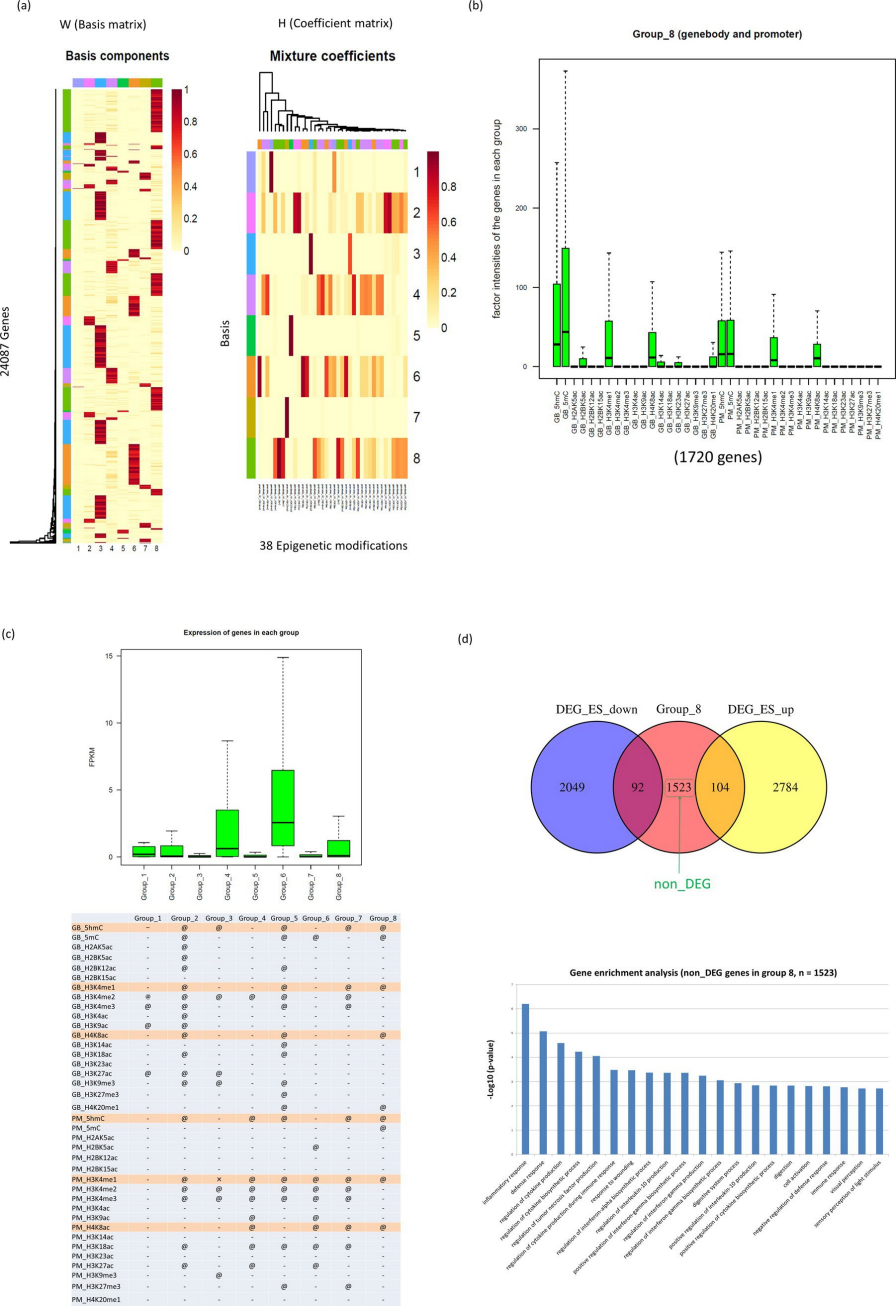
Classification of whole genes and 38 epigenetic modifications by NMF analysis. (a) Whole genes and epigenetic factors (input matrix) were classified into eight groups (the left W-matrix and right H-matrix). (b) Intensities of 19 factors at the gene body and the promoter in group 8 genes. The y-axis shows the distribution of the factor intensities in the genes. GB means gene body and PM means promoter. (c) Expression level of genes in the above mentioned gene groups. The lower figure shows the intensities of whole factors in whole groups. If the intensity of the factor is over 10, the term is designated by “@”. If it is under 10, it is designated by “-”. (d) The result of gene enrichment analysis of non_DEG genes in group 8. The group 8 genes are classified into three groups (DEG_ES_up, DEG_ES_down and non_DEG). The lower figure shows the result of gene enrichment analysis using the genes of non_DEG. The heights of bars indicate the degrees of associations with each term.

To explore the co-occurrence of epigenetic factors more systematically, we applied a nonnegative matrix factorisation (NMF) algorithm [14–16]. Using an input matrix consisting of the intensities (i.e., frequency of occurrences) of 19 epigenetic factors at the genomic features (i.e., gene body and promoter regions) of 24,087 genes, we identified eight groups of genes that are characterised by the co-occurrence of epigenetic factors (Figs 4A and 4B and S6). In addition, the functional aspects of each group were explored through gene enrichment analysis.

We observed the following: (1) group 6 genes, which are marked by H3K4me2, H3K4me3, H3K9ac, and H3K27ac (transcription activation marks) in promoter regions, exhibit active gene expression (Figs 4C and S6) and are also involved in fundamental cellular activities (e.g., RNA processing and chromatin organisation); (2) on the other hand, group 5 genes, which are characterised by H3K27me3 (+) and H3K4me3 (-) in gene body regions, show repressive expression, which may lead to the inactivation of certain developmental processes; (3) interestingly, the promoters of genes in group 7 show both H3K27me3 (repressive mark) and H3K4me3 (activation mark) enrichment and exhibit low expression levels (Fig 4C). The gene enrichment analysis suggests their significant involvement in development. These results are consistent with previous reports [7, 8], indicating that genes marked by bivalent histone domains at promoters are in a poised state during transcription, which is a unique feature in ESCs. Finally, (4) group 8 genes (1720 genes) are significantly enriched in 5hmC, 5mC, H3K4me1 and H4K8ac at both gene body and promoter regions (Fig 4B), supporting our other finding described above (Fig 1). The group 8 genes exhibit relatively moderate levels of gene expression (Fig 4C). Since the gene enrichment analysis failed to show significant enrichment in group 8 genes overall, we classified the genes into the three subgroups used previously (DEG_ES_up, DEG_ES_down, and non_DEG). As a result, we found that the major subgroup, non_DEG group (1523 genes), showed an association with several immunity-related features, such as “inflammatory response”, “regulation of cytokine production”, and “immune response” (Fig 4D). Among them, the “immune response” process included genes associated with both the adaptive and the innate immune system (S2 Table).

### Robustness of our results with different methods

To confirm the robustness of our results in various conditions, we first calculated the correlation in Fig 1 using Spearman’s correlation coefficient instead of Pearson’s correlation coefficient (S7A Fig). Although the overall correlation values were lowered, the values between 5hmC, H3K4me1, and H4K8ac were relatively higher (0.69 for 5hmC and H3K4me1; and 0.38 for 5hmC and H4K8ac).

Next, we compared the results obtained from different peak calling tools (S7B Fig). As shown in the figure, MACS2 also captures peaks detected by HOMER in POU5F1 (Oct3/4), SOX2, and NANOG, which are specifically expressed in ESCs. We conclude that almost the same peaks are captured by both tools in spite of slight differences.

## Discussion

Previous studies have reported that DNA and histone modifications can cooperate with each other to influence gene expression [17, 18]. The bivalent domain at promoters in ESCs is an example of this. We considered the possibility that there may be other interrelationships among DNA and histone modifications that constitute important novel epigenetic codes in the development of the biological nature of ESCs. Since 5hmC is abundant in ESCs [1–3] and is thought to be deeply involved in conferring the properties of pluripotent stem cells, we investigated which of 17 histone modifications and 5mC show high co-occurrence with 5hmC.

Strong correlations were found between several factors; the correlation coefficients among 5hmC, 5mC, H3K4me1, and H4K8ac were particularly high. Although a high correlation between 5hmC and H3K4me1 in hESCs has already been reported [13], as far as we know, that between 5hmC and H4K8ac in ESCs constitutes a novel finding. Acetylation is the only known modification at H4K8; this is observed in transcriptional regulatory regions and is thought to contribute to transcriptional activation [19]. In addition, H4K8ac is suggested to be involved in transcriptional elongation rather than initiation [20, 21]. High correlations among 5hmC, H3K4me1, and H4K8ac were also observed in data derived from different detection methods and species (mouse). Further, the correlations become higher when observed at the gene level. These results imply that these epigenetic factors coexist in groups of genes and influence their expression in ESCs.

Then, we further investigated the relationship between each factor enrichment and gene expression by classifying the whole genes based on DEGs. There were marked differences of the level of each factor between the DEG-based classes at gene region (Fig 2A). In addition, the scatter plots showed that the co-occurrence of the factors was more effective for gene expression than the factors alone (Fig 2B and 2C). These results imply the importance of both 5hmC itself and the trivalent code.

Next, we investigated the correlation between the co-occurrence of these three factors (5hmC, H3K4me1, and H4K8ac) and ESC-specific gene expression. For that, we clustered genes based on the similarity of their ESC-specific intensity patterns for the set of epigenetic factors. As a result, we were able to confirm the importance of their co-occurrence. In Cluster 6, the expression logarithm ratio between hESCs and fibroblasts of genes enriched in 5hmC, H3K4me1 and H4K8ac was large (Fig 3A), while in Cluster 2, the gene expression ratio was small where the intensity ratio for H3K4me1 was small. Similarly, in Cluster 5, the gene expression ratio was small where the intensity ratio for 5hmC was small. The relative activation of Cluster 6 genes in ESCs was most conspicuous (boxplot in Fig 3A). This tendency was confirmed not only at gene body regions but also at promoter regions (Cluster 13 and Cluster 14 in Fig 3B). It should be noted that some of the typical marker genes of pluripotent stem cells (*Nanog* and *Oct3/4*) show this type of co-occurrence. We also noticed that genes of Cluster 18 with the pattern 5hmC (+), 5mC (+), H3K4me1 (-), and H4K8ac (-) show repressive expression in hESCs, although such genes were too few in number (five genes) to interpret the meaning of this finding (S3 Fig).

Next, using the intensities of various DNA and histone modifications, we performed NMF analysis for a systematic search of the co-occurrence of epigenetic factors. The obtained gene group (group 6) was characterised according to fundamental cellular activities by gene enrichment analysis, and group 7 exhibited characteristics of bivalent genes. These results are fully in line with the nature of pluripotent stem cells. Group 8 genes showed significant enrichment of 5hmC, 5mC, H3K4me1, and H4K8ac at the gene body and promoter regions, in agreement with our results that were obtained using a different approach (Fig 1). Hence, these results suggest that group 8 genes influence biological processes in ESCs like bivalent genes.

Gene enrichment analysis showed that the major subgroup (non_DEG genes) of group 8 is enriched in immune-related genes. This may look somewhat inconsistent with the result of the clustering analysis, which showed that co-occurrence of the trivalent factor (5hmC, H3K4me1, and H4K8ac) is found in genes specific to pluripotent stem cells. However, like in bivalent genes, the co-occurrence of epigenetic factors does not always result in significant upregulation or downregulation of expression. As in the signal transduction processes, even low expression of a gene can have significant effects on pluripotency.

Previous studies suggested that ESCs show lower susceptibility to immune rejection and have diminished immunogenicity compared with differentiated tissues [22, 23]. Besides that, *LILRB2*, which belongs to the *LIR* family of genes listed in S2 Table, binds to the MHC class I molecules of antigen-presenting cells and transduces a negative signal that prevents the stimulation of the immune response. Immune response in ESCs could be dependent on the expression of such genes, and their expression could be controlled by the novel epigenetic code. And it seems reasonable that 5hmC, which is mainly present in ESC, is used as a part of the mark. However, further research is needed.

In this study, we used heterologous data obtained from independent experiments with various conditions. Therefore, it may be important to confirm the robustness of our results in various conditions. For that, we confirmed the reproducibility of the results in this study using another peak calling tool (MACS2), and calculation method (Spearman’s correlation coefficients). These results reinforce the robustness of our results.

Since only public data was used in this study, the experimental verification of our results would be indispensable. In addition, the causal relationship among the triplet factors (e.g., which of the DNA and histone modifications precedes the other) is yet to be investigated. Although epigenetic mechanisms in plants are often different with those in animals, there is a related study in *Arabidopsis* [24], in which a histone acetyltransferase is reported to regulate active DNA demethylation. Namely, the histone acetyltransferase, IDM1, binds methylated DNA which lacks H3K4 di- or tri-methylation and acetylates H3K18 and H3K23, leading to DNA demethylation by Repressor of Silencing 1 (ROS1) and others. A similar mechanism can exist in animals. Indeed, in human, there is a protein, Tip60, which recognizes H3K4me1 and acetylates H4K8 [25, 26].

## Conclusion

Our findings suggest that the co-occurrence of three epigenetic factors, DNA hydroxymethylation, H4K8ac and H3K4me1, influences ESC-specific gene expression. Thus, this study proposes a novel epigenetic code which may modulate the biological nature of ESCs.

## Materials and methods

### Data preparation and preprocessing

We downloaded public data for analysis from the Gene Expression Omnibus and the DNA Data Bank of Japan (S1 Table). The data used were on 5mC, 5hmC and histone modifications obtained by hMeDIP-seq [27], MeDIP-seq [28], MBD-seq [29], hMe-Seal [30], and ChIP-seq using immunoprecipitation with antibodies or proteins, TAB-seq [31], and RNA-seq data.

The downloaded SRA data underwent quality control in PRINSEQ software [32], which involved the trimming of low-quality reads and the removal of short reads and adapter sequences. Afterward, reads were mapped to a reference genome (hg19). Bowtie2 [33] was used for hMeDIP-seq, MeDIP-seq, hMe-Seal, MBD-seq, and ChIP-seq data, and Tophat2 [34] was used for RNA-seq data. Bismark [35] was used for TAB-seq data. Multi-mapped reads were removed. Peak calling was performed using HOMER software findPeaks command [36] by setting the FDR threshold to 0.001 for 5mC, 5hmC, and histone modifications mapped reads, and a obtained mapped read count from each peak was normalised as follows:

$$Normalized\;read\;count=\frac{mapped\;read\;count}{Total\;read\;count}\times 1,000,000$$

Each normalised read count was assigned to each gene using HOMER annotatePeaks.pl.

The calculation of read count for RNA-seq data, normalisation and assignment to each gene were also performed using HOMER software.

### Bioinformatic analysis

#### Definition of intensity for epigenetic factors and calculation of correlation coefficient

The normalised read count for each epigenetic factor per gene was taken as the intensity of each factor. Pearson’s correlation coefficients between factors were calculated using those intensities (Fig 1A and 1C).

#### Definition of DEGs and grouping of whole genes based on DEGs

In order to investigate the distribution change of each epigenetic factor (5hmC, H3K4me1, and H4K8ac) due to a change in gene expression, DEGs between human somatic cells (fibroblasts) and hESCs were first identified using TCC (R package; FDR set to 0.05). Among these DEGs, those with more than twice the gene expression in absolute values were adopted. As a result, 5029 DEGs were identified (S2 Fig).

Next, all genes were classified into the following three groups based on these DEGs: genes showing significant upregulation in hESCs (DEG_ES_up), genes showing no significant difference in gene expression in hESCs (non_DEG), and genes showing significant downregulation in hESCs (DEG_ES_down) (Figs 2 and 4D).

#### Patterning of whole genes based on a combination of changes in epigenetic factors

By combining the differences in intensity of 5hmC, 5mC, H3K4me1 and H4K8ac in each gene body region between human fibroblasts and hESCs, whole genes were classified into 19 patterns (for promoter regions, 20 patterns). Next, it was determined which patterns showed a remarkable association with gene expression change. Intensity ratios were expressed as FC in hESCs relative to human somatic cells on a log2 scale (Figs 3A and 3B and S3 and S4).

### NMF analysis

Whole genes were classified into eight groups using NMF (R package). The rank at which the cophenetic value started to decrease was adopted as the optimum factorisation rank (S5 Fig). We used Euclidean distance and a complete method for clustering. Factor intensities of genes in each group were investigated using a boxplot for all eight groups (Figs 4B and S6).

In addition, we investigated the gene expression of all groups (Fig 4C). Finally, gene enrichment analysis was performed using DAVID software [37] for genes included in all groups.

## Supporting information

S1 Fig Correlation coefficients between histone modifications and 5hmC obtained using different detection methods (hMeDIP-seq and TAB-seq) and species (mouse). (TIF)

S2 Fig Definition of DEGs. (TIF)

S3 Fig Combinatorial patterns of 5hmC, 5mC, H3K4me1, and H4K8ac at the gene body. (TIF)

S4 Fig Combinatorial patterns of 5hmC, 5mC, H3K4me1, and H4K8ac at promoter regions. (TIF)

S5 Fig Determination of NMF rank. (TIF)

S6 Fig Factor intensities of genes in each group and gene enrichment analysis. (TIF)

S7 Fig Robustness of our results with different methods. (TIF)

S1 Table Public data used in this study. (XLSX)

S2 Table Genes correlated with ‘immune response’, according to rank 8 gene enrichment analysis. (XLSX)
