# Supplementary figures and images for: A hypothetical trivalent epigenetic code that affects the nature of human ESCs
Source: PLoS One. 2020 Sep 10;15(9):e0238742. doi: 10.1371/journal.pone.0238742 (PMC7482980; doi:10.1371/journal.pone.0238742)

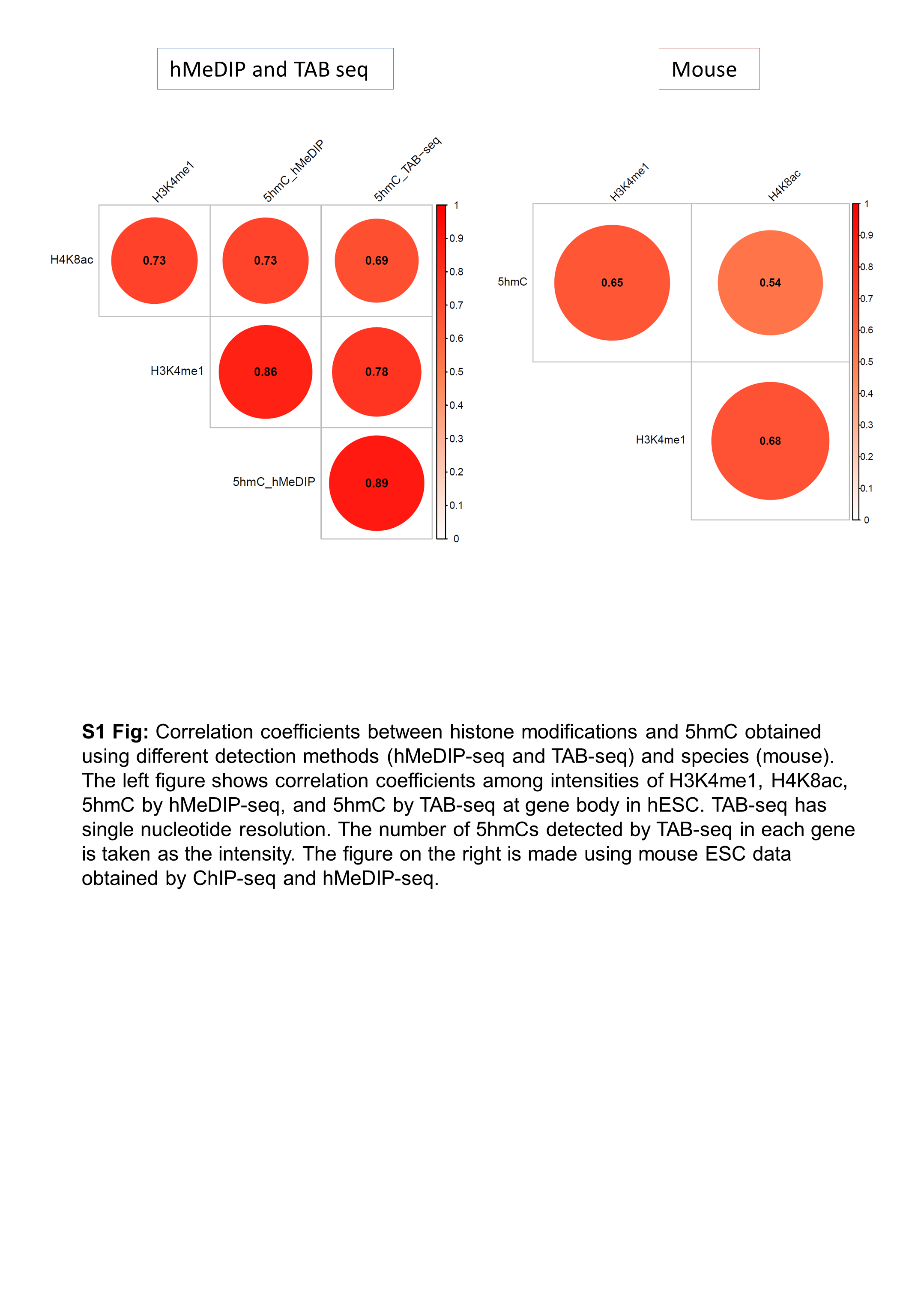

Supplement: S1 Fig — (TIF) [file pone.0238742.s001.TIF]

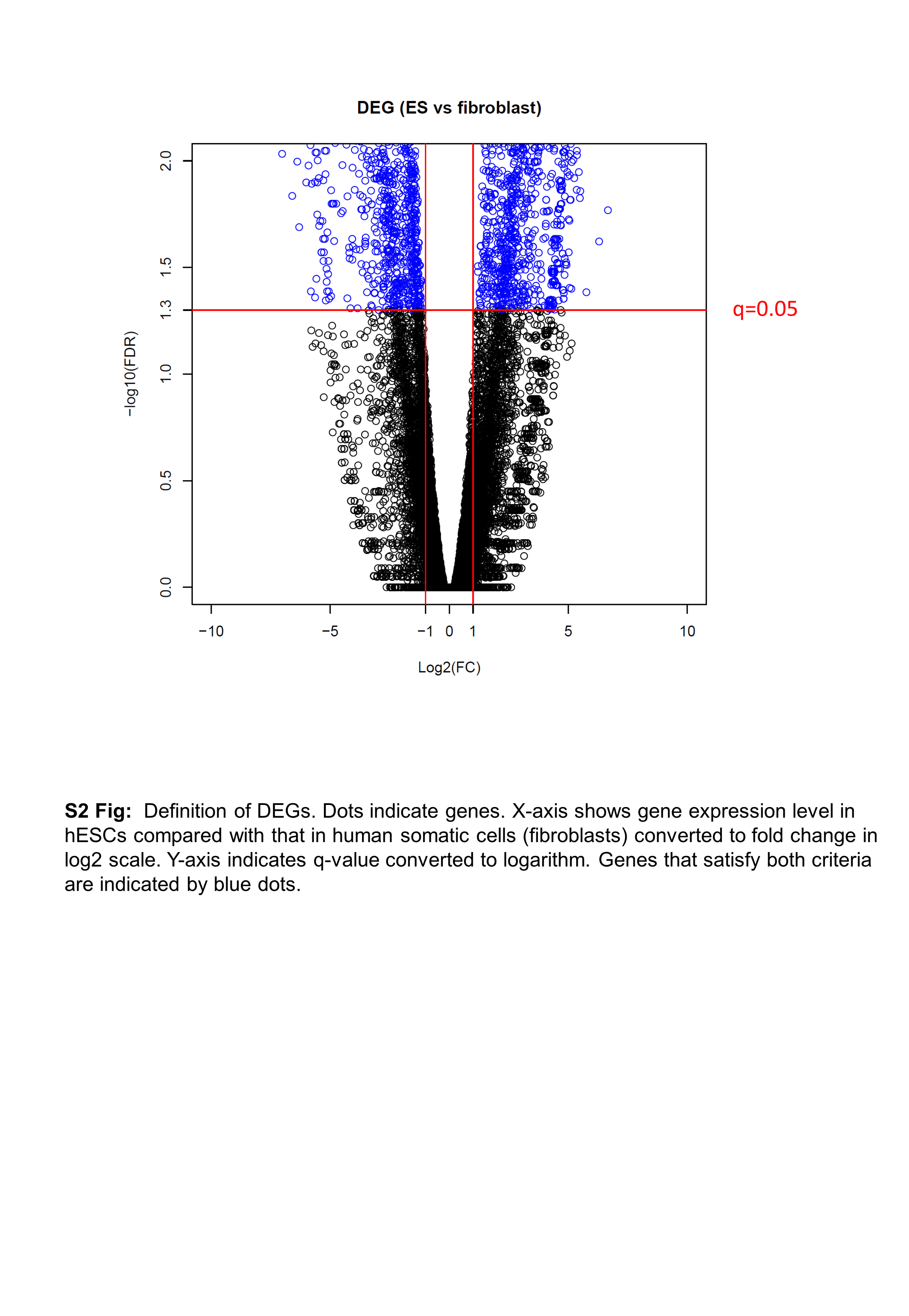

Supplement: S2 Fig — (TIF) [file pone.0238742.s002.TIF]

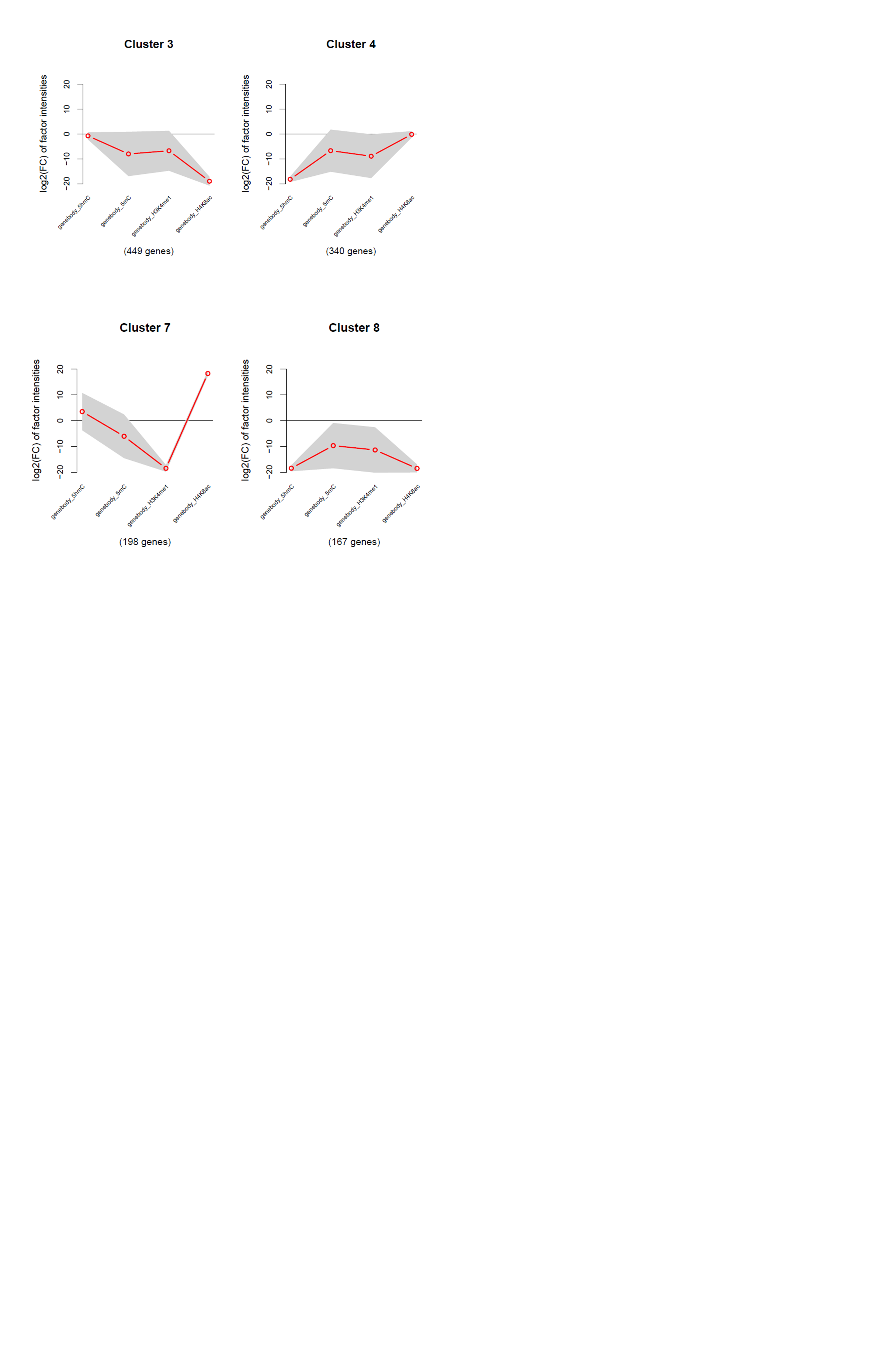

Supplement: S3 Fig — (TIF) [file pone.0238742.s003.TIF]

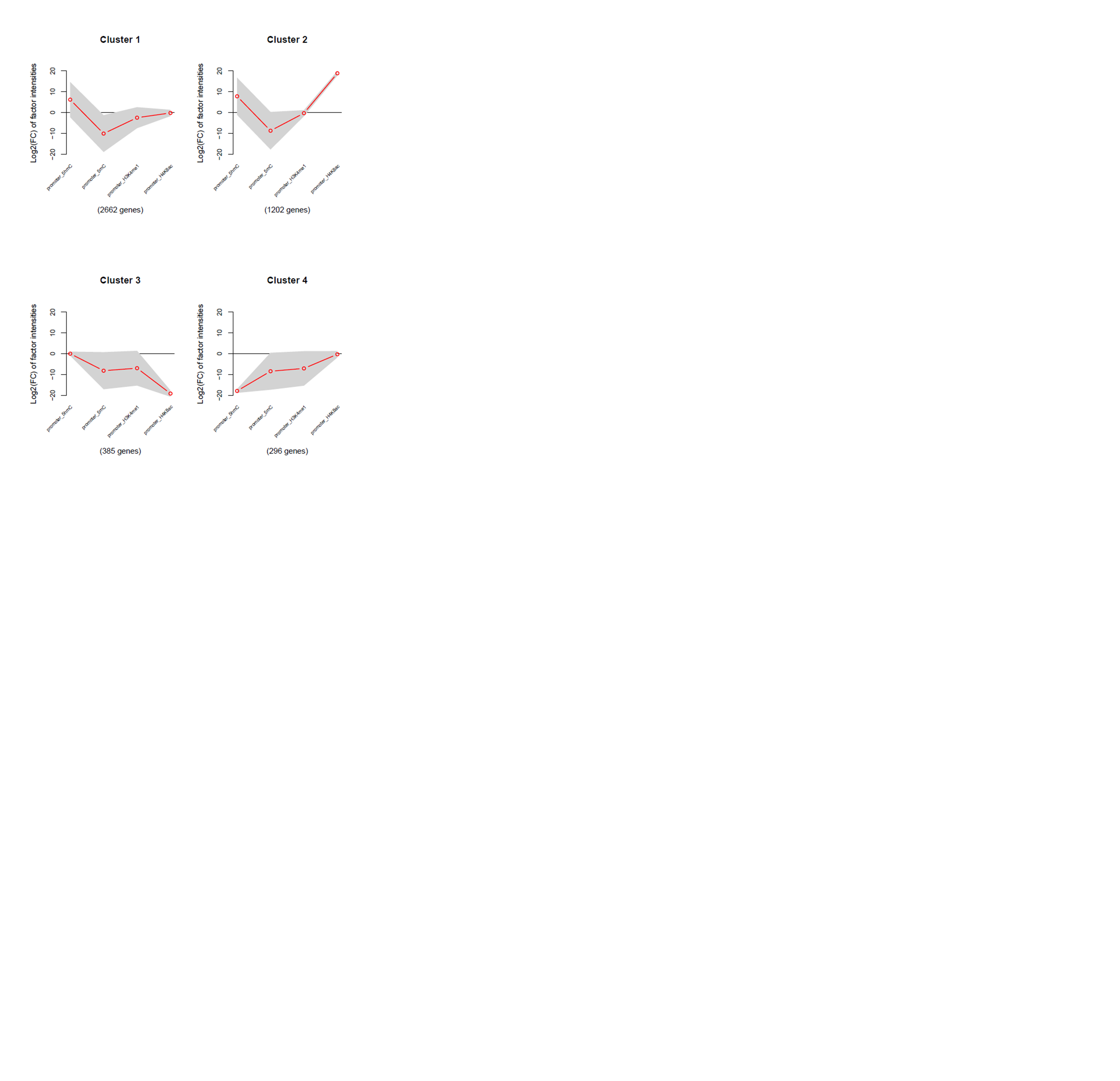

Supplement: S4 Fig — (TIF) [file pone.0238742.s004.TIF]

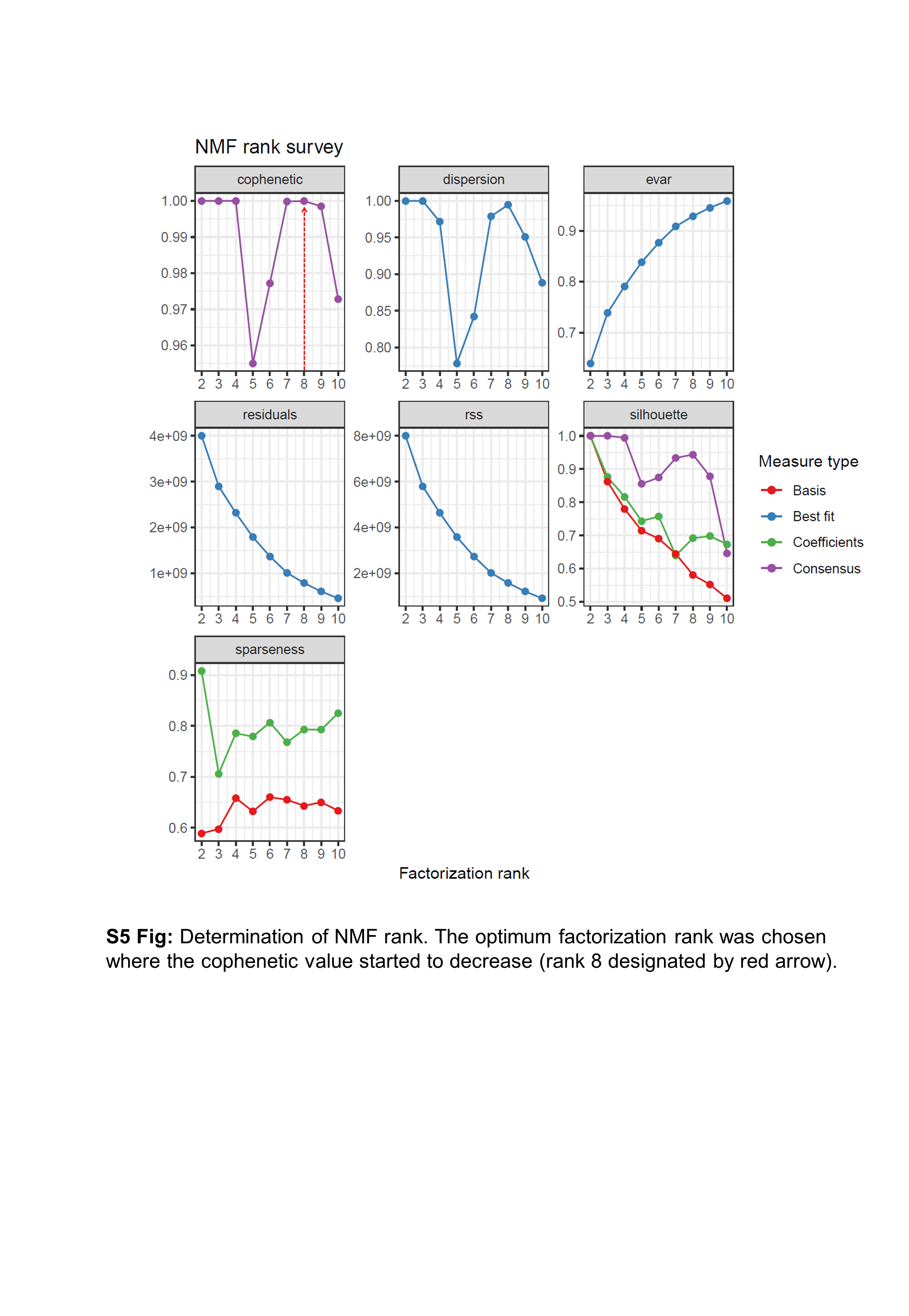

Supplement: S5 Fig — (TIF) [file pone.0238742.s005.TIF]

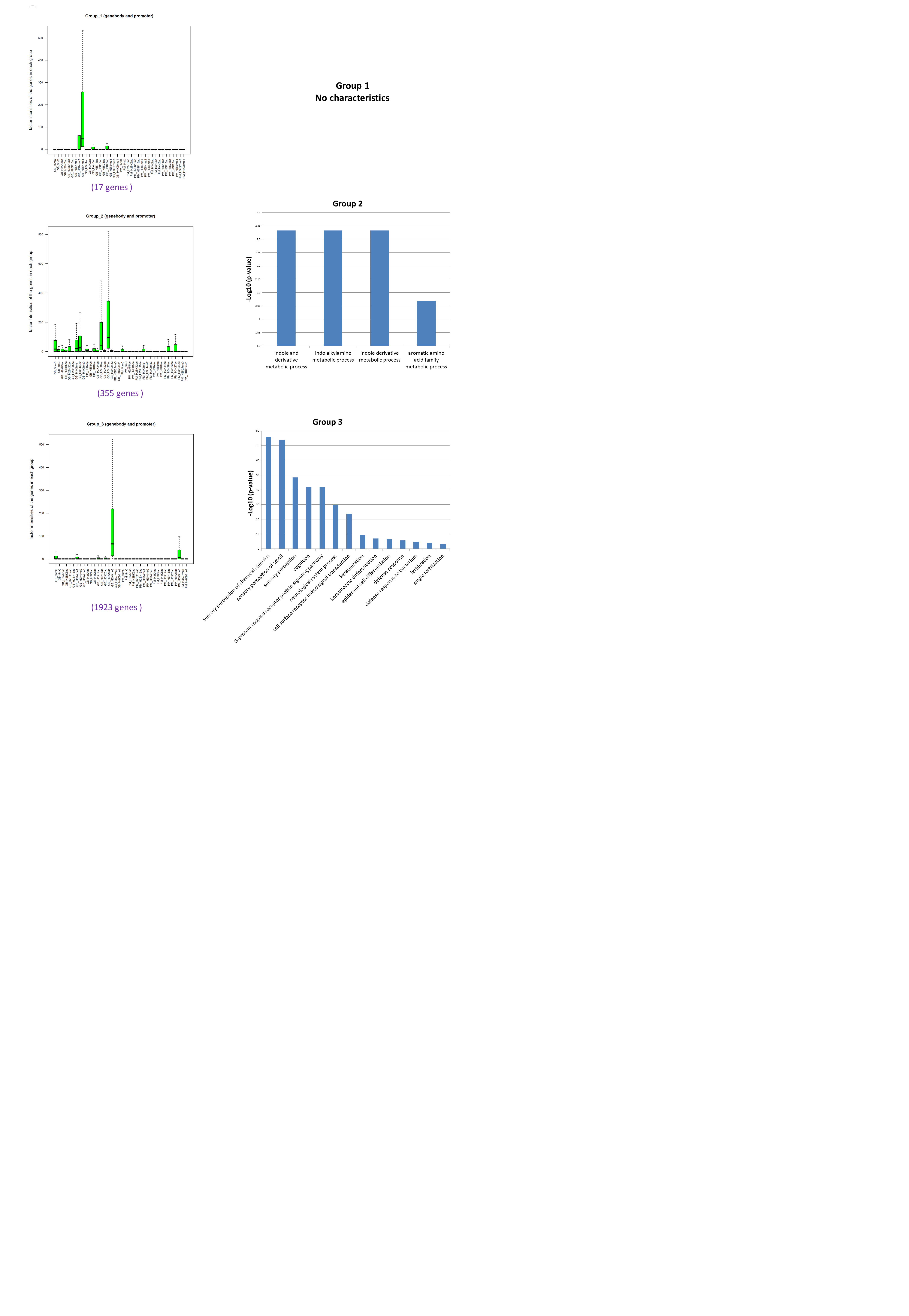

Supplement: S6 Fig — (TIF) [file pone.0238742.s006.TIF]

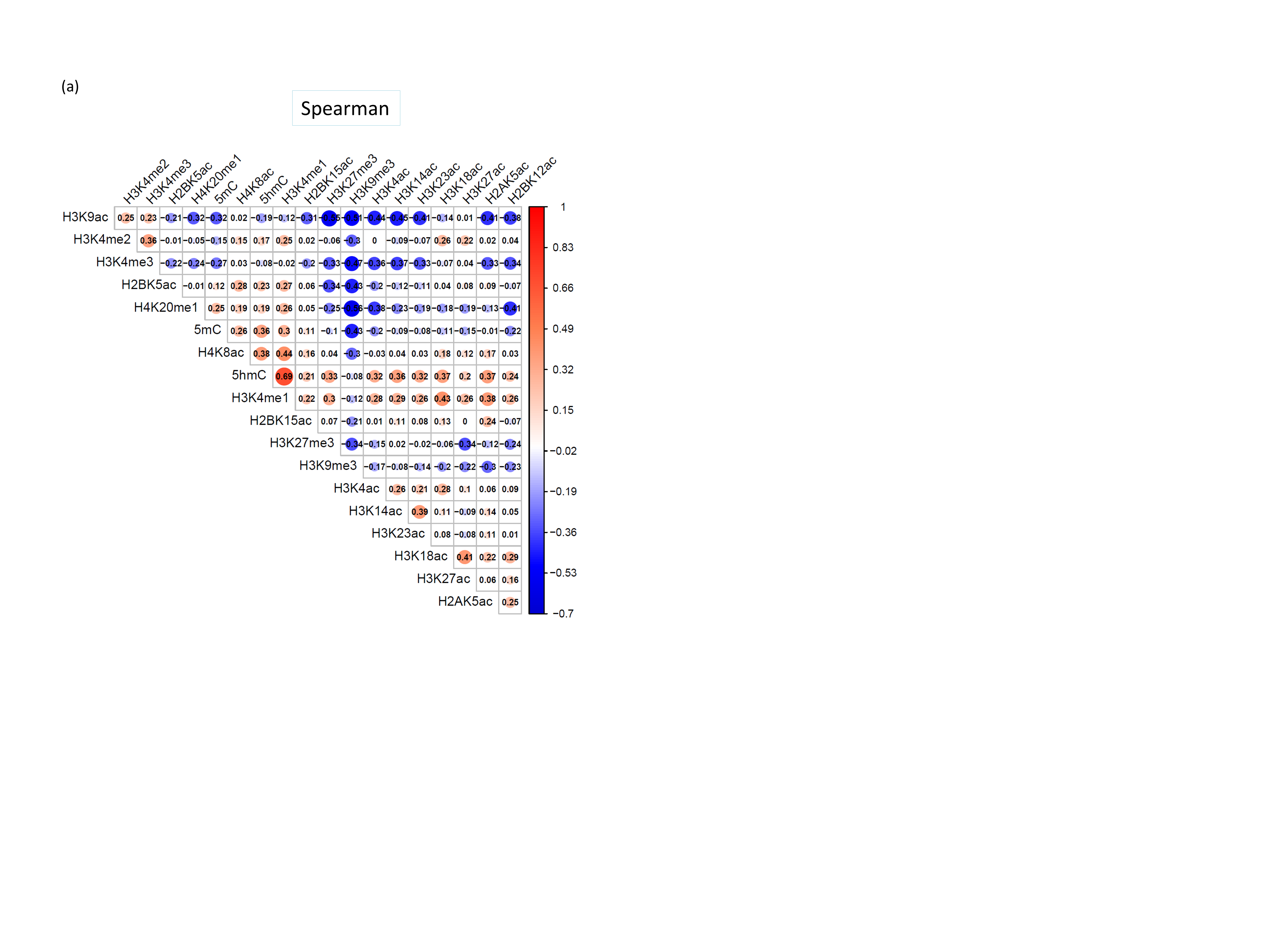

Supplement: S7 Fig — (TIF) [file pone.0238742.s007.TIF]
